# Symptomatic Congenital Cytomegalovirus Infection in Children of Seropositive Women

**DOI:** 10.3389/fped.2017.00134

**Published:** 2017-06-09

**Authors:** Ines Mack, Marie-Anne Burckhardt, Ulrich Heininger, Friederike Prüfer, Sven Schulzke, Sven Wellmann

**Affiliations:** ^1^Pediatric Infectious Diseases, University of Basel Children’s Hospital (UKBB), Basel, Switzerland; ^2^Department of Endocrinology and Diabetes, Princess Margaret Hospital for Children, Perth, WA, Australia; ^3^School of Paediatrics and Child Health, The University of Western Australia, Perth, WA, Australia; ^4^Pediatric Radiology, University of Basel Children’s Hospital (UKBB), Basel, Switzerland; ^5^Neonatology, University of Basel Children’s Hospital (UKBB), Basel, Switzerland

**Keywords:** cytomegalovirus infections, pregnancy, hearing loss, calcification, blueberry muffin, magnetic resonance imaging, neuroimaging

## Abstract

Cytomegalovirus (CMV) is the most frequent congenital virus infection worldwide. The risk of congenital CMV (cCMV) transmission is highest in seronegative women who acquire primary CMV infection during pregnancy. A growing body of evidence indicates that secondary CMV infections in pregnant women with preconceptual immunity (either through reactivation of latent virus or re-infection with a new strain of CMV) contribute to a much greater proportion of symptomatic cCMV than was previously thought. Here, we describe a case of symptomatic cCMV infection in the newborn of a woman with proven immunity prior to pregnancy. Diagnosis was confirmed by CMV PCR from amniotic fluid and fetal MR imaging. The newborn presented with typical cCMV symptoms including jaundice, hepatosplenomegaly, cholestasis, petechiae, small head circumference, and sensorineural hearing loss, the most common neurologic sequela. CMV was detected in infant blood and urine by PCR, and intravenous ganciclovir was initiated and continued orally for 6 weeks totally. Apart from persisting right-sided deafness, the child exhibited normal neurological development up through the last follow-up at 4.5 years. To date, the most effective strategy to prevent vertical CMV transmission is hygiene counseling for women of childbearing age, which, in our case, and in concordance with recent literature, applies to seronegative, as well as seropositive, women. Once an expecting mother shows seroconversion or signs of an active CMV infection, there are no established procedures to reduce the risk of transmission, or therapeutic options for the fetus with signs of infection. After birth, symptomatic infants can be treated with ganciclovir to inhibit viral replication and improve hearing ability and neurodevelopmental outcome. A comprehensive review of the literature, including our case study, reveals the most current and significant diagnostic and treatment options available. In conclusion, the triad of maternal hygiene counseling, postnatal hearing screening of all newborns, followed by CMV PCR in symptomatic infants, and antiviral therapy of infants with symptomatic cCMV provides an outline of best practice to reduce the burden of CMV transmission sequelae.

## Background

Cytomegalovirus (CMV) is a highly prevalent infectious agent in the general population, and seropositivity rates in adult women range from between 40% (in most European countries) and 90% (in most African and Asian countries) (1–3). In the past, symptomatic congenital CMV (cCMV) infection was thought to occur almost exclusively after primary infection of the mother during pregnancy, whereas preexisting maternal CMV immunity was thought to prevent the unborn child from infection in the case of maternal recurrent infection. This suggests that populations with higher seroprevalence rates may have a lower risk of primary maternal CMV infection and, therefore, lower rates of symptomatic cCMV. However, data from populations with low-socioeconomic status and high seropositivity rates in women of childbearing age usually have higher overall rates of cCMV infection (1–2%) compared with the global average (0.4–0.7%) (3). Up to 10% of these infections result in symptomatic congenital disease, in which the same proportion of children will be asymptomatic at birth but will later develop permanent sequelae (1–3).

Currently, the most important strategy to reduce the risk of cCMV infection is hygiene counseling of pregnant women (3). Once an expecting mother shows seroconversion or signs of an active CMV infection, there are no established procedures to reduce the risk of transmission to the child. Similarly, no therapeutic options for the fetus with signs of infection are available. After birth, antiviral treatment in symptomatic infants can decrease the risk of hearing loss, the most common neurological sequelae, and reduce the risk for neurodevelopmental delay in infancy (4). However, strategies aimed at identifying the optimal route and duration of treatment differ widely due to a lack of randomized controlled trials (5).

Our objective, therefore, was to summarize, along with a review of the literature, the latest and most significant diagnostic, treatment, and prevention options prompted by an actual case of symptomatic cCMV in the newborn of a woman with proven immunity prior pregnancy.

## Case Presentation

**Figure 1:**
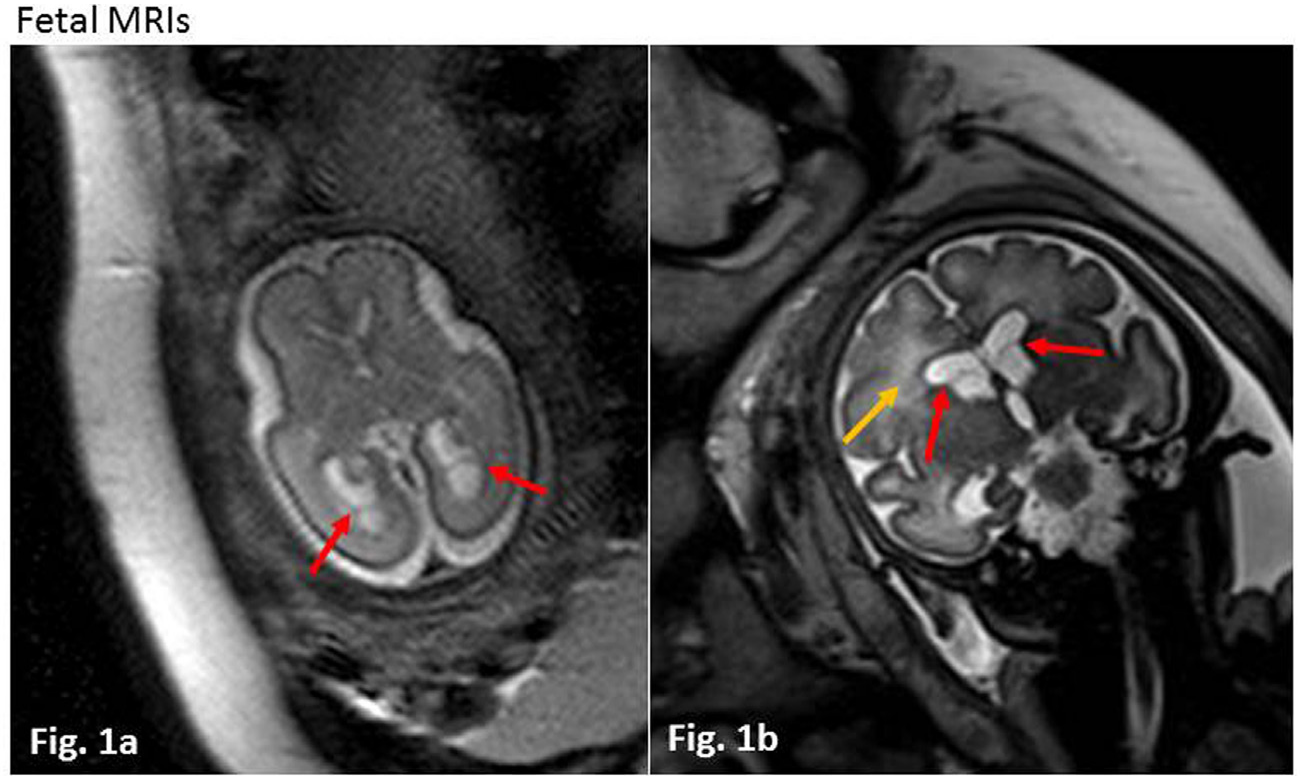
Fetal magnetic resonance imagings (MRIs). **(A)** Week 26 of gestation, axial T2 haste weighted sequence. Red arrows indicate intraventricular cysts in the posterior horns of slightly dilated lateral ventricles. **(B)** Week 36 of gestation, coronar T2 haste weighted sequence. Red arrows show intraventricular cysts, yellow arrow marks diffuse white matter lesions with increased T2 intensity.

We present the case of a term baby girl born to a healthy 38-year-old mother. Pregnancy was uneventful until a fetal ultrasound revealed subependymal cysts at 26 weeks of gestation. Prenatal magnetic resonance imaging (MRI) confirmed bilateral intraventricular cysts and mild dilation of both posterior horns of the lateral ventricles (Figure 1A). At 36 weeks of gestation, a second MRI revealed additional diffuse white matter hyperintensities (Figure 1B). While the mother had documented CMV IgG serum antibodies prior to pregnancy, amniocentesis was performed subsequent to MRI and CMV PCR from amniotic fluid was positive (8.96 log GEq/ml). Therefore, maternal recurrent CMV infection during pregnancy was diagnosed.

The girl was born by spontaneous vaginal delivery at 40 weeks and 1 day gestation. During delivery, meconium staining of the amniotic fluid was noted and Apgar scores were 8, 8, and 10 at 1, 5, and 10 min, respectively. Umbilical cord pH values were 7.20 (arterial) and 7.34 (venous). Signs of mild respiratory distress disappeared at 10 minutes of life, and no further respiratory support was required. Her birth weight was 3,140 g [Percentile (P) 19], length 50 cm (P29), and head circumference was 33 cm (P8). Upon physical examination, extensive petechiae, hepatosplenomegaly, and jaundice were found.

**Figure 2:**
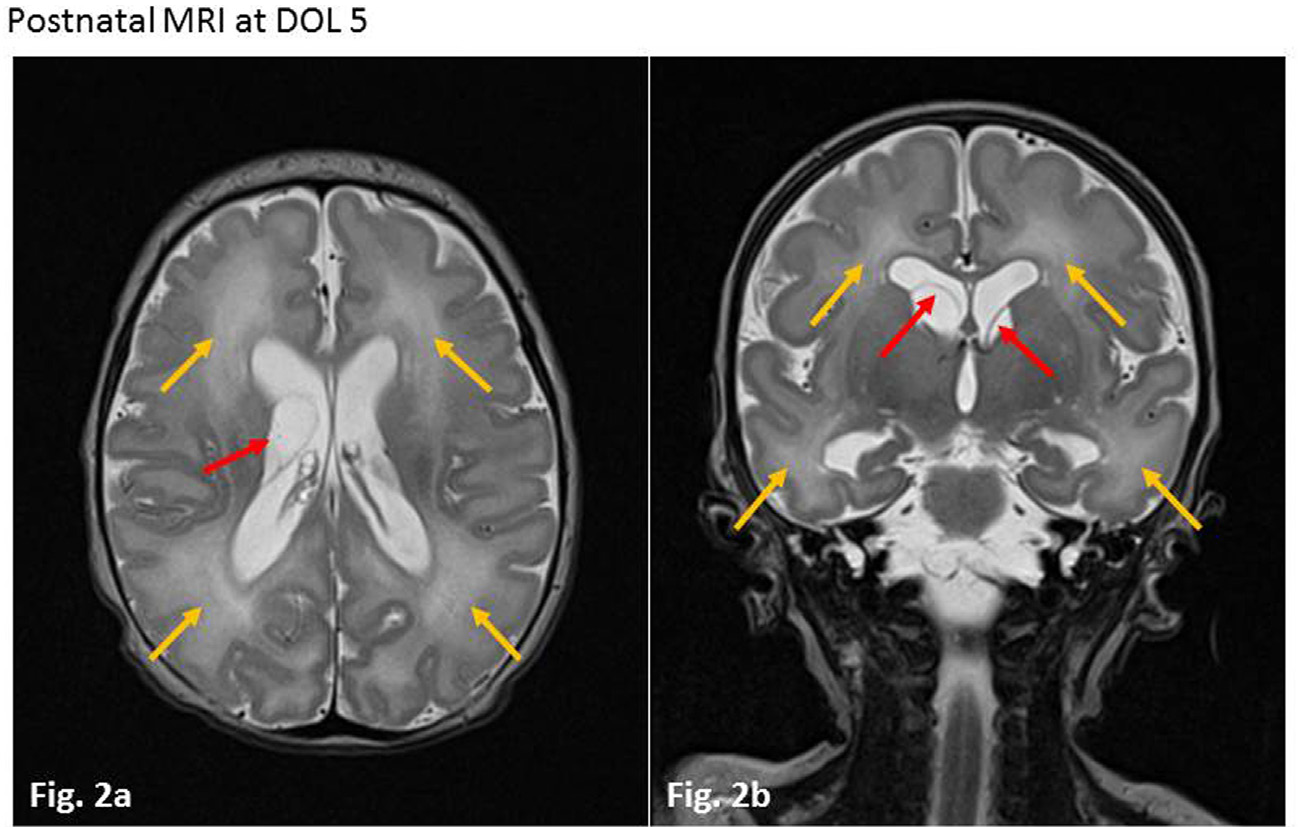
Postnatal magnetic resonance imagings (MRIs) at day of life 5. Axial **(A)** and coronar **(B)** T2-weighted sequences. Red arrows show septa of intraventricular cysts in the area of the former matrix germinativa (loco classico). Slight dilatation of both lateral ventricles. Yellow arrows mark white matter hyperintensities bi-frontally and occipitally. No signs of calcification or polymicrogyria.

Congenital CMV infection was suspected and confirmed within the first days of life by serology (CMV IgG 1,473.4 AU/ml, IgM 0.95 TW), and PCR in infant blood and urine samples (9,710 and 7.09 log GEq/ml, respectively). After informed consent to administer antiviral therapy was obtained from the parents, intravenous (i.v.) ganciclovir (7.5 mg/kg/dose 12 h) therapy was initiated. Postnatal MRI on day 5 of life revealed bilateral subependymal cysts, mild dilatation of both lateral ventricles and white matter hyperintensities (which had already been detected during fetal imaging), but neither calcifications nor polymicrogyria were observed (Figures 2A,B). Severe thrombocytopenia (5 × 10^9^/l minimal count) required repeated platelet transfusions. Profound hepatopathy (ALAT 369 U/l and ASAT 863 U/l maximal count, respectively) led to conjugated hyperbilirubinemia (257.6 µmol/l maximal count), coagulopathy, and progressive cholestasis within the first 2 weeks of life. Otoacoustic emissions and acoustic-evoked potentials, performed on days 4 and 21 of life, were abnormal on the right side. Eye examination was unremarkable except for preretinal bleeding spots and EEG was normal.

Over 3 weeks of i.v. ganciclovir therapy, liver dysfunction improved, and CMV viral load in the plasma decreased significantly, from maximal values of about 10,000 GEq/ml to minimal 1,000 GEq/ml. Therapy was changed to oral valganciclovir (18 mg/kg/dose 12 h), and the girl was discharged with close interdisciplinary follow-up.

The child was regularly seen in our outpatient clinic and viremia persisted with viral loads between approximately 1,000 and 3,000 GEq/ml. Oral valganciclovir was stopped after a total of 6 weeks of antiviral treatment. She exhibited normal neurological development apart from mildly reduced muscle tone and suspected sensorineural hearing loss (SNHL) on the right side. The latter was confirmed by electric response audiometry at the age of 1 month. At the age of 12 months, she still showed normal neurological development. In addition, muscle hypotonia had disappeared, but hearing loss on the right side was still detectable. Unfortunately, no serum viral load was determined.

**Figure 3:**
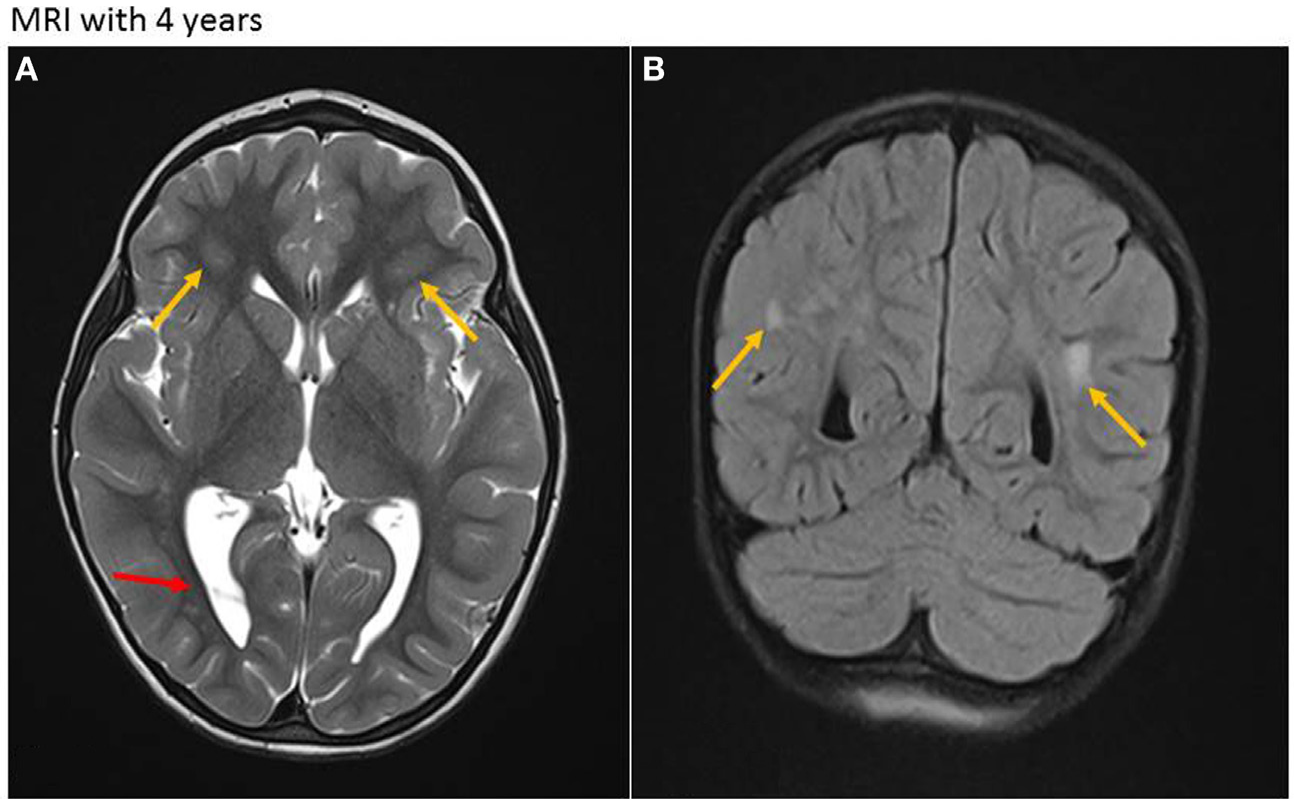
Magnetic resonance imaging (MRI) at age of 4 years. **(A)** Axial T2-weighted sequence. Residual white matter hyperintensities (due to demyelination and gliosis) with periventricular location on both sides (yellow arrows) and persistent intraventricular septa, mainly in the occipital region (red arrow). **(B)** Coronar T2-weighted sequence. Residual periventricular hyperintense white matter lesions (yellow arrows).

At the age of almost 4 years, the girl presented with acute unsteadiness (mild ataxia) at our emergency department. Multiple laboratory examinations, including cerebrospinal fluid and blood tests for viral or bacterial infections revealed negative results. Unfortunately, no CMV diagnostic was made when the child underwent a thorough workup for her disorder. A cranial MRI showed regressive cerebral abnormalities (periventricular white matter lesions on both sides due to demyelination and gliosis, and intraventricular occipital adhesions, Figures 3A,B) without any signs of intracranial bleeding, infection or tumor. During hospitalization, she developed discrete signs of an upper respiratory tract infection while neurological symptoms slightly decreased. Symptoms were interpreted to be of parainfectious origin, and there was no evidence of a relationship with cCMV.

The girl was last seen for follow-up at 4.5 years of age. She still exhibited normal neurological development. As a residuum of cCMV infection, deafness on the right side persisted [hearing threshold level >90 dB, defined as severe hearing loss (4)]. Further auditory follow-up until the age of 6 years was recommended.

## Discussion

The risk for fetal CMV infection is greatest with maternal primary infection and less likely with recurrent infection (3). However, observation of highest birth prevalence rates of cCMV infection in populations with high anti-CMV IgG immunity in women of childbearing age indicates an important role for recurrent CMV infections (3, 6, 7). What needs to be elucidated is whether these are endogenous (due to reactivation) or exogenous (due to infection with a different strain of CMV) instances. Boppana et al. (8) investigated CMV strain-specific IgG in pregnant women and clearly demonstrated that two-thirds of cCMV infections in previously seropositive women were caused by exogenous reinfection. On the basis of the finding that viral isolates from the remaining mother–infant pairs had identical restriction-fragment patterns, it was thought that intrauterine transmission of reactivated CMV was the cause of the cCMV.

Congenital CMV infection is the leading non-genetic cause of SNHL in early childhood, accounting for 21% of children with hearing loss at birth and 24% of those with hearing loss at 4 years of age (4). It can occur immediately after birth in symptomatic CMV infected children, but approximately half of reported cases of hearing loss due to cCMV infection are late-onset and, therefore, cannot always be detected at birth through newborn hearing screening (9). A recent study describes how the timing of seroconversion in primary maternal CMV infection is a strong predictor of postnatal sequelae, with a higher risk when seroconversion occurs in the first trimester rather than later in pregnancy (10).

Availability of prenatal MR imaging has increased in recent years but data regarding sensitivity, specificity, and positive predictive values is still limited. In a recent meta-analysis comparing the diagnostic performance of prenatal ultrasound with prenatal MRI regarding brain abnormalities in general, prenatal MRI clearly outperformed prenatal ultrasound (11). In pregnancies with CMV-proven seroconversion, prenatal prediction of SNHL and neurological impairment by prenatal MRI showed comparable accuracy at the end of the second or in the third trimester, with a high negative predictive value. It has been shown that MRI and the time of onset of seroconversion in pregnancy are independent predictors of postnatal SNHL, and that only MRI is an independent predictor of neurological impairment (10). Furthermore, ventriculomegaly and calcifications were estimated as non-specific findings for CMV and accordingly were not graded separately (10).

In our patient, maternal anti-CMV IgG serum antibodies were documented prior to pregnancy. Maternal recurrent CMV infection was diagnosed following detection of fetal brain abnormalities in a routine ultrasound screening during pregnancy (Table 1A), and two prenatal MRIs were performed at 26 and 36 weeks of gestation. They demonstrated intraventricular cysts and periventricular white matter lesions with increased T2 intensity resembling a very common finding in cCMV infection, associated with good postnatal prognosis.

**Table 1:** Criteria for diagnosis, therapy and follow-up of cCMV.

**(A) Interpretation of CMV serology in pregnancy**					
**Indications for screening**	**CMV antibodies**	**IgG avidity**	**Interpretation**	**Implications**	**Group**
– As a part of the diagnostic evaluation of flu-like illness in pregnancy – When a fetal anomaly suggestive of cCMV infection is detected on prenatal ultrasound examination	IgG− IgM−	n.a.	Uninfected or early infection	Counsel about behavioral measures to reduce risk of acquiring infection	1
	IgG− IgM+	n.a.	May be false positive due to other virus infections	Repeat tests in 2 weeks	2
	IgG+ IgM−	High	Past infection	Counsel about low risk of fetal infection and possible sequelae.Every trimester of pregnancy: – CMV viral load – CMV IgG, IgM Absence of a significant rise in serial IgG titers or viral load suggests absence of reactivation or reinfection	3
	IgG+ IgM+	High	Past or recurrent infection	Counsel about low risk of fetal infection. Possible sequelae if fetus is infected.Every trimester of pregnancy: – CMV viral load – CMV IgG, IgM A significant rise (at least 2-fold) in serial IgG titers suggests reactivation or reinfection	4
	IgG+ IgM+	Low	Recent infection	Counsel about likelihood of fetal infection, possible sequelae, and options for prenatal diagnosis and management.Every trimester of pregnancy: – CMV viral load – CMV IgG, IgM	5
**(B) Diagnosis, therapy and follow-up in cCMV infection**					
**Indications for screening**	**Diagnostic approach**	**Therapeutic approach**	**Long-term follow-up**		
– Newborns with abnormal hearing screening test (OAE): retest, if failure again: ABR and screen for CMV – Maternal seroconversion during pregnancy (see group 3 and 4 if significant titer rise, and group 5)	Birth to 3 weeks of age: – CMV PCR (urine > saliva) 3 weeks to 1 year of age and/or to discriminate between congenitally and postnatally acquired infection: – CMV PCR (dried blood sample, “Guthrie test”; if not feasible, testing urine and/or saliva for CMV by PCR, or measurement of CMV IgG serum antibody)	– 0–28 days: ganciclovir 6 mg/kg/dose intravenously 12-hourly (adjusted in neonates with renal failure); 1 month to 18 years: 5 mg/kg/dose intravenously 12-hourly (1) – 0–28 days: valganciclovir 16 mg/kg/dose orally 12-hourly (if clinically stable and able to take oral medications, usually after 2–6 weeks of intravenous therapy); 1 month to 18 years: 520 mg/m^2^/dose (max. 900 mg) 12-hourly (1)	– Hearing assessments every 6 months until 3 years old, then annually until 6 years old		
– Infants with clinical symptoms typical for cCMV infection	If positive: CMV DNAemia: – Viral load in whole blood (qPCR) Evaluation of organ involvement: – Physical, neurologic, and neurodevelopmental examination, including measurements of weight, length, and head circumference – Complete blood count with differential count, coagulation studies, liver function tests, renal function tests – Hearing evaluation – Ophthalmologic evaluation – Neuroimaging	– Treatment duration total: 6 months – Treatment response: • Regular general physical examination • Neurological examination • Hearing evaluation every 3–6 months • Ophthalmologic evaluation every 3–6 months (more frequent in infants with chorioretinitis) • CMV viral load in whole blood or plasma, frequency depending on severity of illness – Treatment goal: undetectable or near undetectable CMV DNAemia level before stopping treatment	– Ophthalmologic assessments annually until 5 years old – Regular dental visits		

*n.a., not applicable; ABR, auditory brainstem response; OAE, otoacoustic emissions; cCMV, congenital cytomegalovirus; CMV, cytomegalovirus*.

Treatment options for cCMV infections are still limited. Available drugs inhibit CMV replication but cannot eliminate the virus from the human organism. After discontinuation of antiviral treatment, an increase of the viral load in blood is frequently observed (4). Studies have shown that a 6-week course of ganciclovir, especially when started during the neonatal period, is effective in terms of decreasing the severity of neurological dysfunction and hearing loss in symptomatic and asymptomatic infants (12–14). Oral valganciclovir is more easily administered to infants with cCMV infection (15, 16), and results in plasma concentrations are similar to those obtained when using ganciclovir (17). It was suggested that the initial benefit of a 6-week course of ganciclovir could wane over the first years of life (4). Therefore, a randomized, placebo-controlled trial in neonates with symptomatic cCMV disease was recently performed, comparing 6 months with 6 weeks of valganciclovir therapy. The results indicate that long-term vs. short-term treatment is associated with moderately improved long-term audiologic and neurodevelopmental outcomes, with no significant differences in the rate of adverse events (4). Regarding viral load in whole blood, virus copies similarly decreased in the two study groups during the first 6 weeks of treatment and then showed an increase in the group receiving short-term treatment. Reduced viral loads correlated with better hearing outcomes at 6, 12, and 24 months among participants in the 6-month treatment group, whereas no such effect was observed in the 6-week treatment group (4). Beneficial effects of extended treatment (up to 12 months) were also shown in observational studies (18, 19).

In our case, the patient (born before the effect of long-term treatment had been demonstrated) was treated with i.v. ganciclovir for 2 weeks, followed by 4 weeks of oral valganciclovir therapy. Last follow-up was performed at 4.5 years of age. At that time, the girl presented with normal neurological development but showed persisting unilateral sensorineural deafness.

Progress toward the development of a vaccine for cCMV has been slow, and the availability of a CMV vaccine is still several years away. Among others, one reason might be the public perception and lack of awareness of CMV. The early identification of CMV-attributable cases, and their successful treatment, is often hampered by the later appearance of damage in a high proportion of children who were either symptomatic or asymptomatic at birth. More sensitive screening methods for CMV infection have been developed in recent years (20), but routine CMV serology screening programs in pregnant women are not established in most countries. However, the beneficial aspects of CMV screening programs before and during pregnancy, respectively, are quite controversial given that neither prevention of vertical CMV transmission in seropositive women nor treatment of the fetus with signs of CMV infection is possible right now. On the other hand, the appearance of clinical symptoms is not a reliable diagnostic tool for cCMV, as only a small percentage of children present with symptoms at birth. For example, hearing loss is often late-onset and therefore cannot always be detected at birth. When cCMV is suspected, the best method of screening today is PCR amplification of viral DNA extracted from neonatal dried blood samples, which are used in many countries to screen newborn infants for metabolic and genetic diseases (Table 1B). No specific samples need to be taken for cCMV screening, which saves costs and eases the implementation of screening in maternity units. Studies comparing DNA and PCR amplification from Guthrie tests with virus isolation in urine specimen (the “gold standard”) showed 99% sensitivity and specificity (21).

While awaiting effective vaccines and improved antiviral drugs, preventative strategies must be based on educating clinicians and women of childbearing age about the mode of CMV transmission and the critical importance of basic hygiene, which is known to decrease the rate of maternal seroconversion or reinfection in seropositive women, respectively (22, 23). Additionally, medical education should help increase awareness of flu-like symptoms in pregnant women (Table 1A).

## Concluding Remarks

A growing body of evidence indicates that exogenous CMV reinfection during pregnancy contributes to a much greater proportion of symptomatic cCMV than previously assumed. As in our case, many women and even physicians are not aware of the risk for recurrent infection during pregnancy, despite preexisting immunity. Therefore, avoiding exposure of pregnant women to CMV through behavioral changes should be recommended for seronegative, as well as seropositive, pregnant women (primary prevention). The conundrum of exogenous reinfection versus endogenous reactivation requires further research.

In our patient, fetal pathologies were detected by ultrasound and confirmed by fetal MRI early in pregnancy. Neuroimaging plays an important role in screening for brain lesions in suspected or confirmed cCMV. In the absence of antenatal treatment options, postnatal hearing tests have the highest significance for screening and should be performed with complete coverage and meticulous follow-up (secondary prevention, Table 1).

Antiviral therapy of symptomatic cCMV infections, regardless of severity, clearly improves hearing ability and neurodevelopmental outcome when commenced in the first weeks of life. However, our patient showed persisting deafness as neurologic sequelae, although antiviral therapy had been administered on the first day of life. Due to possible side effects of antiviral therapy and the complexity of the disease, patients should be treated by experienced teams in specialized centers. Future research should address pending problems regarding the optimal route and duration of administration of antiviral drugs.

Together, our case underscores that, to date, the triad of maternal education, postnatal hearing screening of all newborns followed by CMV PCR in symptomatic infants, and antiviral therapy of infants with symptomatic cCMV provides best practice to reduce the burden of CMV transmission sequelae.

## Ethics Statement

This article reports patient data that have been collected as part of routine clinical practice, with parental consent obtained for the presentation and publication of the clinical case and case report.

## Author Contributions

Conception or design of the work: IM, M-AB, SW, and UH. Data collection: M-AB and FP. Data analysis and interpretation: IM, M-AB, FP, SW, SS, and UH. Drafting the article: IM and M-AB. Critical revision of the article: UH, SW, and SS. Final approval of the version to be published and agreement to be accountable for all aspects of the work in ensuring that questions related to the accuracy or integrity of any part of the work are appropriately investigated and resolved: all authors.

## Conflict of Interest Statement

The authors declare that the research was conducted in the absence of any commercial or financial relationships that could be construed as a potential conflict of interest. The reviewers, GS and CS, and the handling editor declared their shared affiliation, and the handling editor states that the process nevertheless met the standards of a fair and objective review.

## Abbreviations

ABR, auditory brainstem response; (c)CMV, (congenital) cytomegalovirus; NPV, negative predictive value; P, percentile; PPV, positive predictive value; SNHL, sensorineural hearing loss.
